# Percutaneous irreversible electroporation for breast tissue and breast cancer: safety, feasibility, skin effects and radiologic–pathologic correlation in an animal study

**DOI:** 10.1186/s12967-016-0993-7

**Published:** 2016-08-05

**Authors:** Sheng Li, Fei Chen, Lujun Shen, Qi Zeng, Peihong Wu

**Affiliations:** 1 Department of Medical Imaging and Interventional Radiology, State Key Laboratory of Oncology in South China, Collaborative Innovation Center for Cancer Medicine, Sun Yat-sen University Cancer Center, 651 Dongfeng Road East, Guangzhou, 510060 Guangdong People’s Republic of China; 2 Department of Ultrasonography, The Second Affiliated Hospital of Guangzhou Medical University, Guangzhou, People’s Republic of China

**Keywords:** Irreversible electroporation, Breast, Imaging, Pathology, Apoptosis, Skin

## Abstract

**Background:**

To study the safety, feasibility and skin effects of irreversible electroporation (IRE) for breast tissue and breast cancer in animal models.

**Methods:**

Eight pigs were used in this study. IRE was performed on the left breasts of the pigs with different skin–electrode distances, and the right breasts were used as controls. The electrodes were placed 1–8 mm away from the skin, with an electrode spacing of 1.5–2 cm. Imaging and pathological examinations were performed at specific time points for follow-up evaluation. Vital signs, skin damage, breast tissue changes and ablation efficacy were also closely observed. Eight rabbit models with or without VX2 breast tumor implantations were used to further assess the damage caused by and the repair of thin skin after IRE treatment for breast cancer. Contrast-enhanced ultrasound and elastosonography were used to investigate ablation efficacy and safety.

**Results:**

During IRE, the color of the pig breast skin reversibly changed. When the skin–electrode distance was 3 mm, the breast skin clearly changed, becoming white in the center and purple in the surrounding region during IRE. One small purulent skin lesion was detected several days after IRE. When the skin–electrode distance was 5–8 mm, the breast skin became red during IRE. However, the skin architecture was normal when evaluated using gross pathology and hematoxylin-eosin staining. When the skin–electrode distance was 1 mm, skin atrophy and yellow glabrescence occurred in the rabbit breasts after IRE. When the skin–electrode distance was ≥5 mm, there was no skin damage in the rabbit model regardless of breast cancer implantation. After IRE, complete ablation of the targeted breast tissue or cancer was confirmed, and apoptosis was detected in the target tissue and outermost epidermal layer. In the ablated breasts of the surviving animals, complete mammary regeneration with normal skin and hair was observed. Furthermore, no massive fibrosis or mass formation were detected on ultrasound or through hematoxylin–eosin staining.

**Conclusions:**

After IRE, the skin architecture was well preserved when the skin–electrode distance was ≥5 mm. Moreover, breast regeneration occurred without mass formation or obvious fibrosis.

**Electronic supplementary material:**

The online version of this article (doi:10.1186/s12967-016-0993-7) contains supplementary material, which is available to authorized users.

## Background

In recent years, the use of breast-conserving therapy has been supported by clinical evidence, and image-guided ablation techniques, such as radiofrequency ablation [1, 2] and high-intensity focused ultrasound [3], have emerged as useful modalities for the treatment of breast tumors. During breast cancer ablation, if a lesion is too large or is superficial, breast skin can become damaged, and incomplete ablation might occur in an attempt to prevent such damage. In addition, many Asian women exhibit a relatively small breast volume, and thus the risk for skin injury during thermal ablation is increased in this population [4].

Thermal ablation causes a breast tumor to undergo coagulative necrosis. During this process, the original ablation area is gradually substituted postoperatively by fibrous tissue, creating a mass in the breast [5, 6]. This mass might result from inflammation, fibrotic formation, tumor recurrence or fat necrosis. If a breast mass develops, residual tumors must be excluded, which can exacerbate the patient’s psychological burden [1].

Radiation-induced fibrosis after breast-conserving therapy (i.e., fibrosis of breast and skin) is a common late toxicity after radiotherapy. Approximately 45 % of patients have experienced radiation-induced fibrosis-related pain, and more severe fibrosis might be induced after resection of tissues with radiation-induced fibrosis [7]. This is a major factor affecting quality of life and cosmetic results after radiotherapy for breast cancer [8–10].

After breast cancer ablation, if the skin has not been damaged and normal breast tissue has regenerated without mass formation or a residual tumor, then the above- mentioned problems would be resolved. The application of irreversible electroporation (IRE) makes this goal possible, as apoptosis of the skin can be selective after IRE. There have been no reports examining the use of IRE for ablation of breast cancer in a large animal model, although some efforts have been made [11, 12]. The previous studies [11, 12] mainly reported the efficacy of IRE, but we focused on skin damage, fibrotic mass formation and mass reabsorption in the breast after IRE ablation.

In this study, we performed IRE on two large animal breast and breast cancer models, with electrodes placed close to the skin surface (1–8 mm). We evaluated the safety and feasibility of IRE, particularly regarding its potential to damage skin and induce fibrotic mass formation in the breast.

## Methods

Approval for the use of animals in this study was obtained from the Animal Care Committee of Sun Yat-sen University Cancer Center. Wuzhishan pigs were used to study IRE ablation on normal breasts, and New Zealand great white rabbits were used to investigate IRE ablation of breast skin and breast cancer. A description of the design of the study is shown in Table 1.

**Table 1:** Study design and observation items

	Aim	Skin electrode distance/electrode spacing	Observation items	Subgroups
Pig model	To test irreversible electroporation ablation of the breast	3 mm in 1 pig, 5–8 mm in five pigs/electrode spacing: 1.5–2 cm	Safety and feasibility–pathological changes and imaging changes: apoptosis assays, skin and breast regeneration	IRE group: 6 pigs sham treatment group: 2 pigs. Three left breasts in each pig were ablated or processed, and the right breasts were used as controls
Rabbit model	To test irreversible electroporation ablation of the breast with thin skin	1 mm in 4 ablation zones and 3 mm in another 4 ablation zones Electrode spacing: 1.5–2 cm	Safety and skin architecture	8 sessions of IRE performed in 4 rabbits
Rabbit model with breast cancer	To test irreversible electroporation ablation of breast cancer with thin skin	1, 3 mm and no less than 5 mm Electrode spacing: 1.5–2 cm	Changes in pathology and ultrasound: skin architecture, contrast-enhanced ultrasound and elastosonography	4 rabbits Two breast cancer models were generated in each rabbit

The pig breast is relatively large and has thick skin, which facilitated the accurate determination of the approximate distance between the electrode and the skin when IRE was performed.

The skin over a local breast cancer tumor can be different from typical healthy skin; this thinning might result from malnutrition, skin invasion and local tumor tension. Thus, the risk of thermally damaging the skin during IRE can increase. Because rabbit skin is thin, we used New Zealand great white rabbits to study skin damage after IRE. To accomplish this, we generated a VX2 tumor model in rabbits to examine the safety of IRE ablation of breast cancer. Dr. Sheng Li and Dr. Qi Zeng were responsible for performing follow-up examinations to identify breast skin changes in these animals. Before and after IRE, the breast skin, nipple, muscle behind the breast, mammary duct and mammary glands were assessed in all of the animals via gross pathology and histopathology, and mass formation in the breast, fibrosis in the ablation zone and breast regeneration were also assessed post-treatment.

### Irreversible electroporation

All of the animals were fasted for 12 h prior to the procedure. Skin preparation, sterilization and draping were completed before the procedure. Each procedure began with intramuscular Telazol pre-medication (4.4 mg/kg), and after intubation, general anesthesia was maintained via intravenous injection of 3 % pentobarbital sodium (1 ml/kg). All of the animals were subcutaneously administered 0.01 mg/kg buprenorphine before the procedure and every 12 for 72 h thereafter as needed. Each animal also received succinylcholine (2 mg/kg) during IRE. Pancuronium (0.15 mg/kg) was administered intravenously 10 min before IRE to reduce muscle contractions.

IRE was guided under computed tomography (CT) as well as ultrasound to ascertain the precise spacing between the skin and the electrode. An experienced technologist in the medical imaging department performed the CT and Magnetic Resonance Imaging (MRI) scans. Dr. Peihong Wu, a professor of radiology and oncology with 30 years of experience in diagnostic imaging, and Dr. Sheng Li, with 7 years of experience in medical imaging and interventional radiology, analyzed the CT images together. Dr. Fei Chen, with 7 years of experience in ultrasound, performed the ultrasounds and analyzed the images with another trained doctor in the Department of Ultrasonography.

**Fig. 1:**
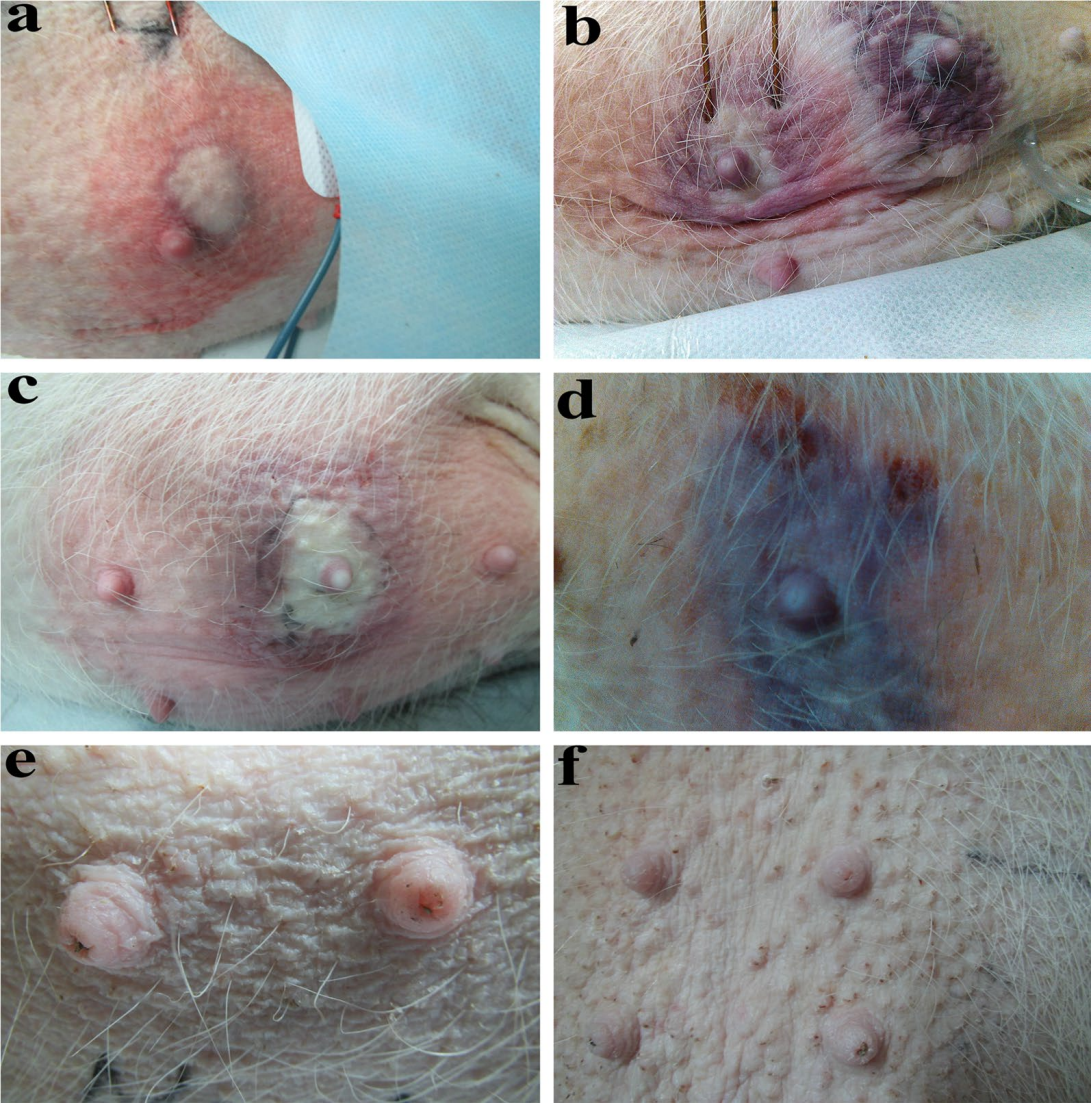
Breast skin changes after IRE. **a**–**c** (skin–electrode distance: 5 mm): Color changes in breast skin with different electrode distances. The skin initially turned *red* (**a**), then *purple* (**b**), and finally became white in the central zone (**c**) near the electrode. **d** IRE ablation performed very close to the superficial skin (skin–electrode distance of 3 mm). The skin was still purple and swollen after IRE. **e**, **f**: 2–3 months after IRE, the breast appeared normal

IRE electrodes were implanted into the proper position in the breast with real-time ultrasound monitoring (MyLab GOLD Platform, ESAOTE S.P.A., Italy). Our group has over 5 years of experience in interventional radiology. The nipple was located between two electrodes, and most of the breast was located in the ablation zone in our study. Parallel electrodes and a proper location were also confirmed by CT scanning (Fig. 1a). After complete muscle relaxation, we began IRE ablation using the electrocardiogram (ECG) synchronous mode.

A NanoKnife^®^ system (software version 2.2.0, Angiodynamics^®^, Latham, NY, USA) was used with the following parameters: two electrodes, ECG synchronization, 90 pulses, a pulse length of 70 µs, an electrode spacing of 1.5–2 cm, 1500 V/cm, and a tip exposure of 1.5 cm. After IRE, we reviewed the voltage and current graphs and the contrast-enhanced ultrasound and CT images to assess the ablation efficacy. If complete ablation of the targeted zone was not achieved, the IRE procedure was repeated.

### IRE ablation of pig breasts

Eight healthy mature Wuzhishan pigs, 16–18 kg, were divided into an IRE group (six pigs) and a sham treatment group (two pigs). Three left breasts in each pig were ablated or processed. The right breasts were used as controls; these received no treatment in any of the pigs. Electrodes were inserted into the left breasts without performing ablation in the sham treatment group. IRE ablation was performed on the left breasts in the IRE group.

In the IRE group, the distance between the electrode and the skin was 3 mm for three breasts in one pig and between 5–8 mm in the remaining five pigs. The distance between the electrode and the skin was defined as the shortest distance between the exposed part of the electrode and the skin surface as measured with CT imaging.

In the sham treatment group, the electrode was inserted into a predetermined position in the breast for the same time interval without performing ablation. CT and MRI were performed perioperatively, and we either performed a biopsy or resected the breasts for pathological examination. Resection was performed immediately prior to an animal being sacrificed.

### IRE ablation of rabbit breasts

To determine whether IRE ablation would damage thin skin, we placed electrodes close to the breast skin (1 or 3 mm) in New Zealand great white rabbits. The electrodes were maintained parallel to the skin plane, and the distance was 1 mm in four ablation zones and 3 mm in another four ablation zones. A total of eight sessions of IRE were performed on four rabbits. At 1, 3, 7 and 14 days after IRE, Dr. Sheng Li and Qi Zeng followed up to examine mass formation, fibrosis and skin changes.

### IRE of a breast cancer model

We sectioned fresh VX2 tumors into 1–2 mm diameter tissue blocks without necrosis or liquefaction pieces. Next, we implanted a fresh tumor under the breast skin of each of four rabbits. Two breast cancer models were generated in each rabbit. The skin–electrode distance was <8 mm.

After 1 week, all 8 breast tumors increased to 1–1.5 cm in diameter, and we assessed tumor size using ultrasound. Color Doppler ultrasound, elastosonography and contrast-enhanced ultrasound were performed before and 1 and 7 days after IRE. We resected four breast tumors from four rabbits on the third day for pathologic examination; thus, no ultrasounds were performed on these resected tumors at later time points in the experiment. The remaining 4 breast tumors were regularly assessed for at least 1 month.

We used contrast-enhanced ultrasound to assess whether complete ablation was achieved, and elastosonography was used to detect changes in tissue stiffness after IRE, as a potential method to assess complete ablation. For elastosonography (Surpersonic Aixplorer color Doppler ultrasonic diagnostic apparatus, Supersonic Shear Imaging Company, France), the probe frequency was 4–15 MHz. After an appropriate section was selected, we switched to the real-time shear wave elastography model and placed an elastic imaging sampling frame over the ablation zone. When image stabilization was achieved after 3–5 s, we filled 90 % of the imaging sampling frame with color and selected a cycle of 3 cm in diameter as the measurement area to obtain the average elasticity value. Measurements were repeated three times. Leaving an ultrasound probe impression on the surface skin was avoided as much as possible. For contrast-enhanced ultrasound imaging, after a bolus injection of 1.2 ml microbubbles of sulfohexafluoride (SonoVue^®^, Bracco, Milano, Italy), we started approximately 2 min of imaging. The ultrasound frequency used was 3–9 MHz. Elastosonography and contrast-enhanced ultrasound were performed before and after IRE.

### Hematoxylin-eosin staining and immunohistochemistry

Two pathologists, each with more than 4 years of experience in our cancer center, performed all of the pathological examinations. Breast specimens were harvested and fixed for HE staining and apoptosis assays. TdT-mediated dUTP Nick-End Labeling (TUNEL) and caspase-3 tests were performed immediately and 1, 3 days and 1 week after IRE to assess apoptosis.

The major reagents used were an In Situ Cell Death Detection Kit, POD (Hoffmann-La Roche Inc. USA), a Caspase-3 polyclonal antibody for IHC (Thermo Fisher Scientific Inc. Rockford, IL US), and a 3,3′-diaminobenzidine and Streptavidin peroxidase immunohistochemistry staining kit (Fuzhou Maixin Biotechnology Development Co., Ltd).

TUNEL and caspase-3 tests were used to detect markers of apoptosis. Positive TUNEL results were defined as brown-yellow nuclear staining. Positive staining for caspase-3 protein was brown-yellow staining of cytoplasm. We assessed the apoptotic cell distributions in the skin, mammary vessels, muscle, mammary gland, duct and stroma.

## Results

### IRE ablation of pig breast

After IRE at a skin–electrode distance of 3 mm, the color of the skin on the pig breasts initially changed to red, then white, then purple, and finally returned to a normal color (Fig. 1a–d). After 2 weeks, there were small suppurative foci in one area of skin. Scabs formed 4 weeks later, and the skin then returned to normal. The remaining two breasts were normal.

When the skin–electrode distance was approximately 5–8 mm, the breast skin became red, and the central area of the ablation zone became white with a red color around the nipple. A closer skin–electrode distance led to more obvious and rapid intraoperative skin color changes. The skin color returned to normal after 2 h, and a small amount of liquid was visible as the puncture point became red.

After IRE, the ablated breasts became congestive, swollen and hard. Within 3 months, the majority of the animals had breast skin that appeared normal (Fig. 1e, f), with intact hair and skin. No fibrous mass formation was detected in the ablation zone during the 3 months following IRE.

### IRE ablation of rabbit breast and breast cancer

**Fig. 2:**
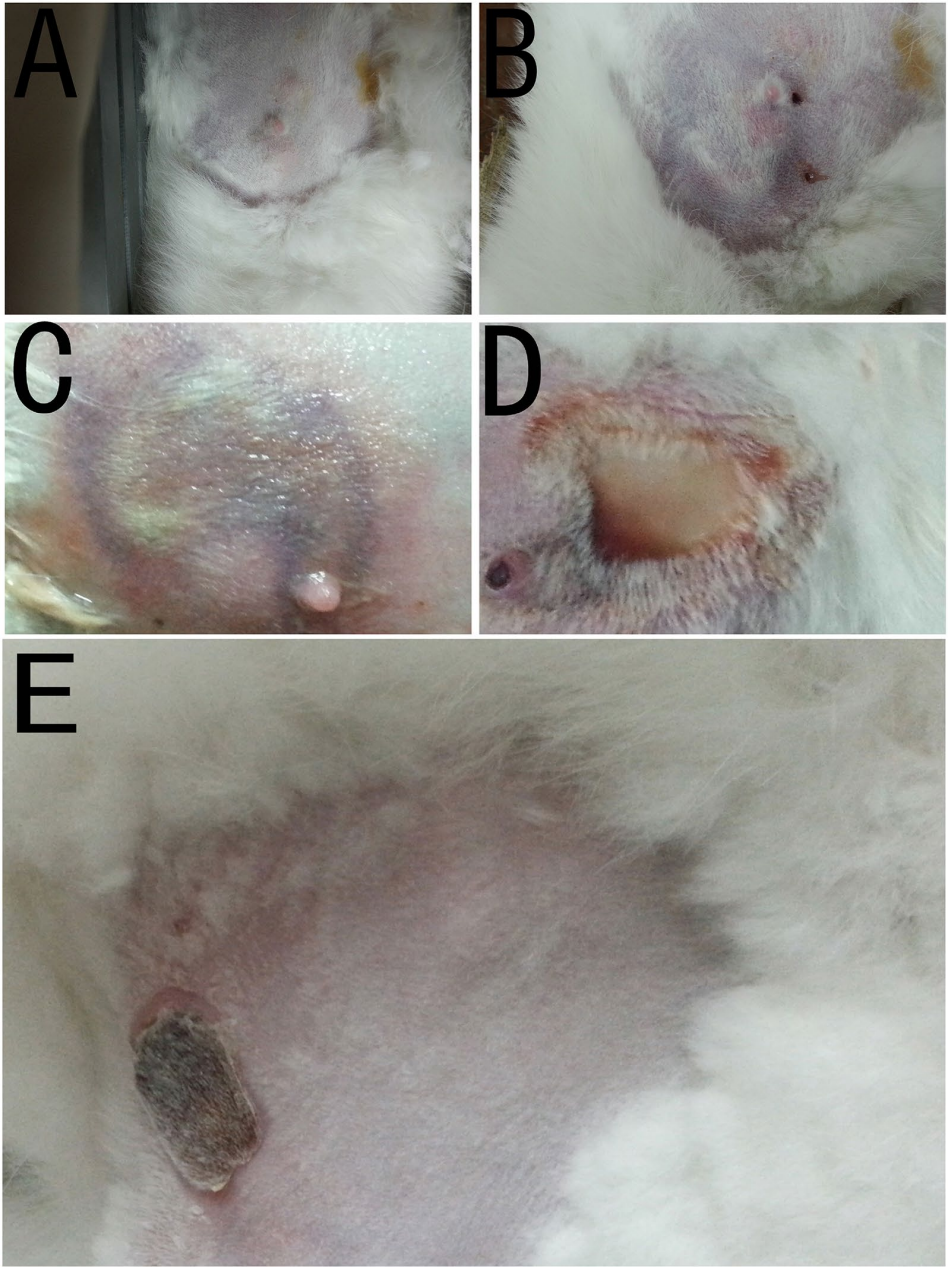
Skin changes after IRE (skin–electrode distance of 1 mm). **a** Before IRE, the breast skin appeared normal. **b** Immediately after IRE, the skin became *red* and *edematous*. **c** Three days after IRE, the skin was still *red* in color. **d** Seven days after IRE, the hair had fallen out, but no suppurative foci were observed in the skin folds. **e** Skin scar remaining after IRE

Before IRE, the breast skin on the rabbits appeared normal (Fig. 2a). When the skin–electrode distance was 1 mm, the skin became red after IRE (Fig. 2b, c), and one small purulent lesion appeared on the third postoperative day (Fig. 2d). One week later, the skin exhibited chlorosis and shedding (Fig. 2e), and 2 weeks after IRE, new skin had regenerated, without scar formation. Three weeks later, only a small scab was present on the skin; new hair growth was evident in this area, and most of the skin in the ablation zone had recovered to normal. Two months after IRE, the breast tissues that surrounded the skin and muscle were normal. With regards to the other three breasts, the skin became dry and yellow, and trichomadesis occurred 1 week later. After 2 weeks, there were no suppurative foci, but new skin had grown, and 3 weeks later, the hair and skin were normal. When the skin–electrode distance was 3 mm, the skin color of four ablation zones transiently changed to red, without skin architecture damage or hair loss, and there was no scar or fibrosis formation in any of the rabbit breasts, which became swollen after IRE.

**Fig. 3:**
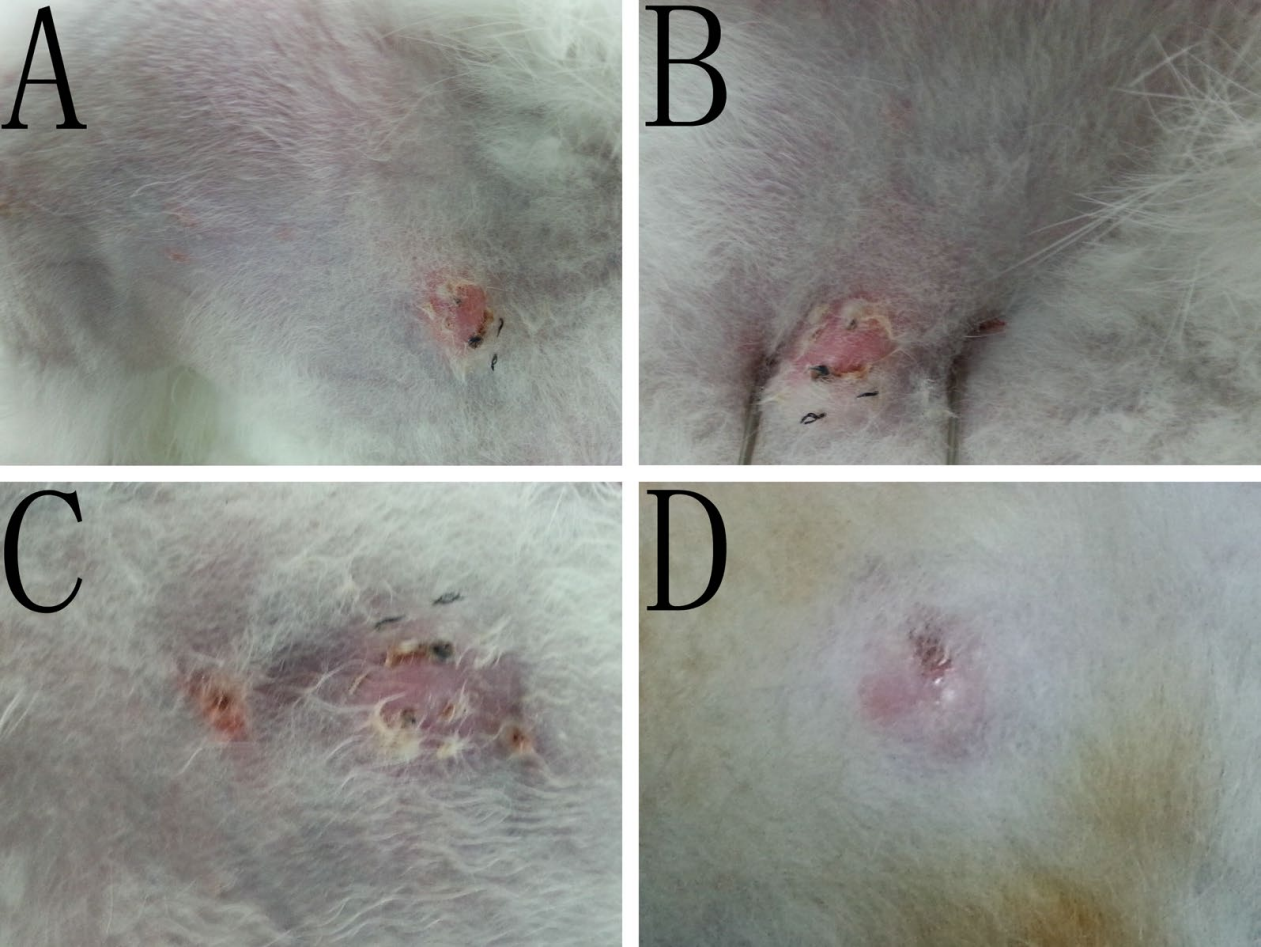
IRE ablation of breast cancer. **a** A breast cancer model was constructed. **b** Two electrodes were placed into the tumor. **c** Immediately after IRE, the skin became *red*. **d** Two weeks after IRE, complete ablation of the tumor was confirmed, and the skin appeared normal with few hairs

After the breast VX2 tumor model was established in rabbits, we confirmed tumor formation using pathological assessment and ultrasound. The tumors gradually increased by 1–2 cm (Fig. 3a) in diameter 10 days after implantation, without liquefaction or necrosis. The first day after IRE (Fig. 3b), the breast tumors became hardened and enlarged, and after the second day, the tumors began to soften and shrink in size in a time-dependent manner. When the skin–electrode distance was 1 mm, the skin on one breast exhibited signs of sepsis with scar healing. When the skin–electrode distance was approximately 3 mm, the breast skin covering the tumor became purulent, and no blood supply was detected in the ablated area. When the skin–electrode distance was ≥5 mm, there were no signs of skin ulceration, suppuration or trichomadesis (Fig. 3c), and the intact skin architecture was well preserved. One month after IRE, all of the animals had breast skin that appeared normal (Fig. 3d).

### Hematoxylin-eosin staining

**Fig. 4:**
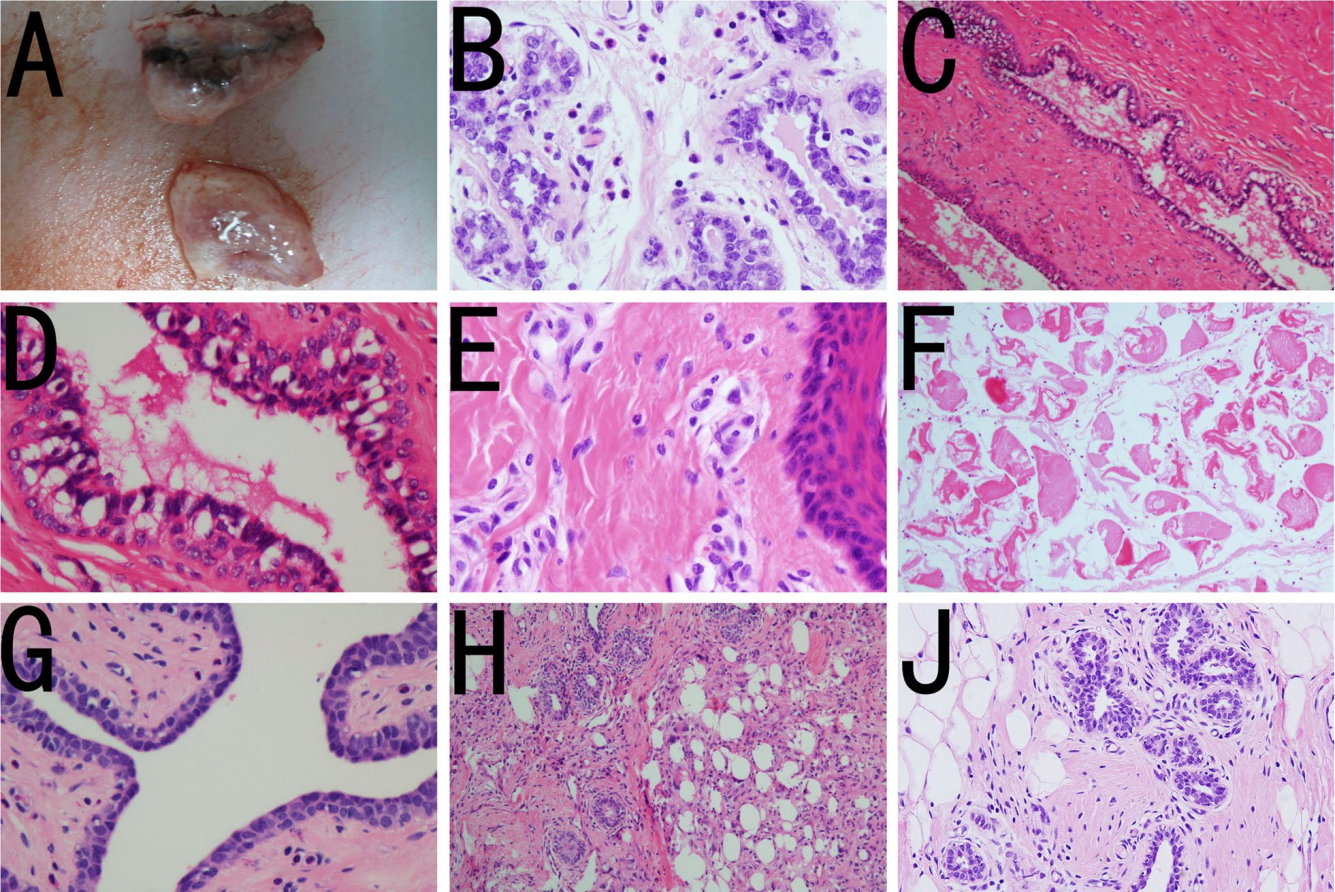
Pathological changes to pig breast after IRE. **a** Gross pathology. The tissue near the electrode tract had darkened, whereas the tissue far from the electrode appeared normal. **b** (×200): vacuolization in breast acinar epithelial cells with normal mammary gland architecture. **c** (×200). **d** (×400) Three days after IRE, there was serous fluid in the mammary duct, with exfoliation of epithelial cells and vacuolization, and the tissue architecture was normal. **e** Squamous cells in the breast skin (*upper figure* ×400) were stained with HE and appeared normal. **f** Damage to nearby striated muscle, with myolysis. **g** (×400) Ten days after IRE, the mammary duct was normal. **h** (×100) Ten days after IRE, collagen fibers formed in the electrode path. **j** (×100) Two months after IRE, the breast tissue (acinar, duct) was normal

Immediately after IRE, the ablated breasts became congested, swollen and hard. Surrounding the electrode tract, the breast color turned brownish-black (Fig. 4a). Immediately after IRE, vacuolation was observed in the glandular and ductal cells of the breasts. Two hours to 3 days after IRE ablation, there was secretion of serous fluid from the breast ducts, shedding of some ductal cells and infiltration of neutrophils, eosinophils and plasmocytes (Fig. 4b–d). No visible damage to the stratified squamous epithelium architecture of the breast skin was observed (Fig. 4e). Three days after IRE, fibroblasts and collagen formation were visible near the electrode path, and striated muscle fragmentation and dissolution were also visible (Fig. 4f). One week after IRE, the sebaceous glands and hair in the skin within the ablation zone appeared normal (Fig. 4g–j).

Fourteen days after IRE, mammary gland regeneration and restoration of normal breast tissue architecture without ductal narrowing, dilatation or occlusion were observed in the pig breasts (Fig. 4g). There was no massive fibrosis or mass formation in any of the ablation zones.

**Fig. 5:**
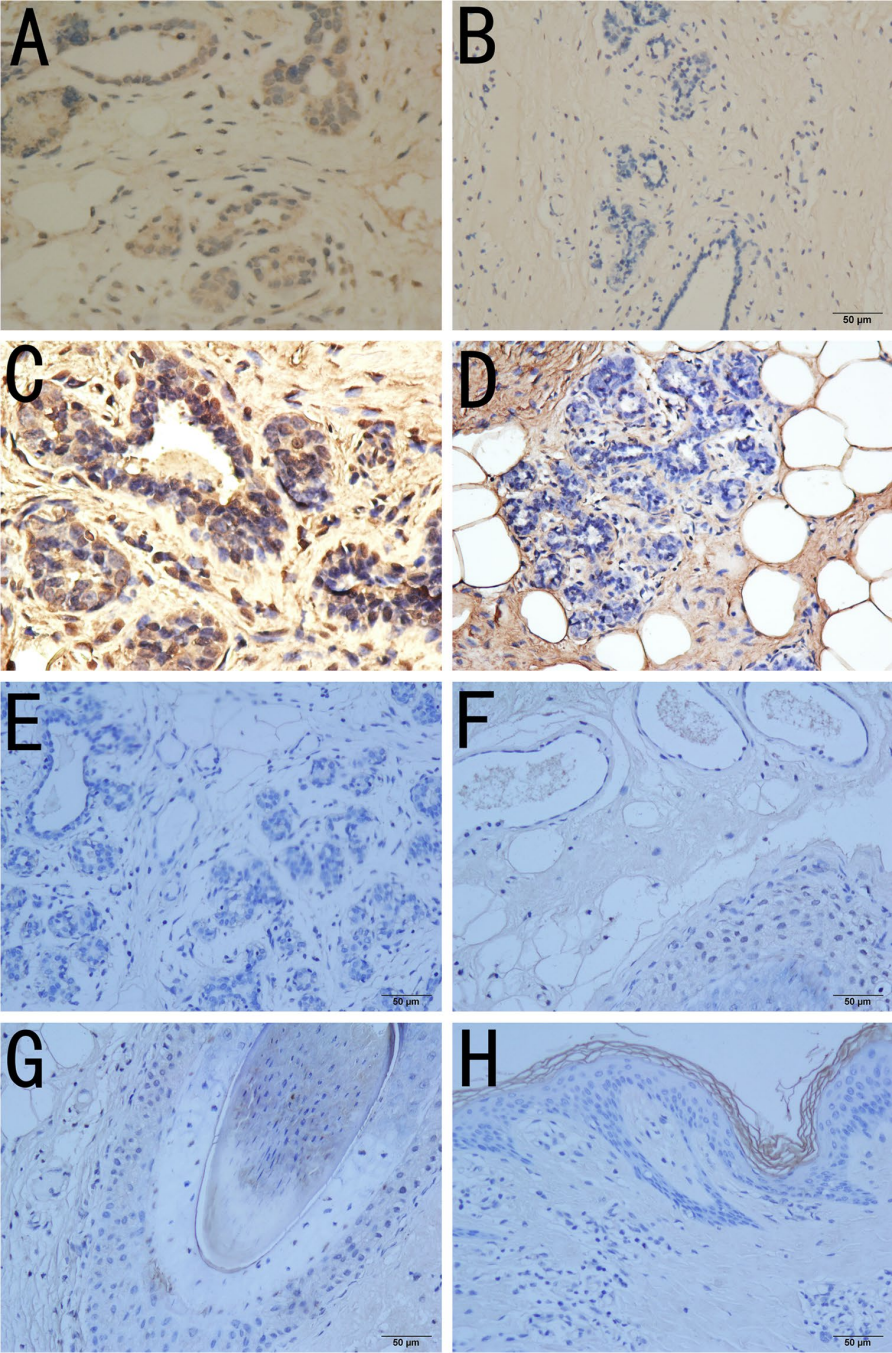
After IRE, apoptosis in the breast was detected. **a** (×200) Caspase-3 immunohistochemistry and cytosolic staining in ducts and alveoli. **b** (×200) Cytosolic staining was negative in the control group. **c** (×400) One day after IRE, the TUNEL assays indicated that most nuclei stained positive, indicating cell apoptosis. **d** (×400) No apoptosis was observed in the control group (*black arrow*). **e** (×400) One week after IRE, the breast tissue was normal. **f** One week after IRE, the architecture and glandular epithelial cells of the sebaceous glands and sweat glands were normal. **g** The hair roots in the breast skin were normal. **h** (×200) One week after IRE, the squamous epithelial cells in the breast skin were normal

A small amount of bleeding in the electrode path was found in both the sham treatment group and the control group, with some collagen fibers forming 3 weeks later (Fig. 5) without detectable apoptosis or inflammatory infiltration.

Two months after IRE, normal breast tissue was present in the previous ablation zones on the experimental pigs (Fig. 4j), and no architectural abnormalities were observed in the skin collagen, hair roots, sweat glands or sebaceous glands. Over a period of 3 months, there was no mass formation or mammary duct occlusion.

For the IRE ablation of normal rabbits, when the skin–electrode distance was within 1–3 mm, HE staining showed clean and intact collagen fibers and normal skin architecture. A homogenously red-stained area around the electrode passage was also observed. No fibrosis or mass formation was observed in any of the breasts 2 weeks after IRE.

After performing IRE ablation of breast tumors, infiltration of inflammatory cells and a few erythrocytes was observed in the skin. The ablated breast tumors gradually resolved after 3 weeks without mass formation, and the previous ablation zones were replaced with normal breast tissue.

### Apoptosis assays

**Fig. 6:**
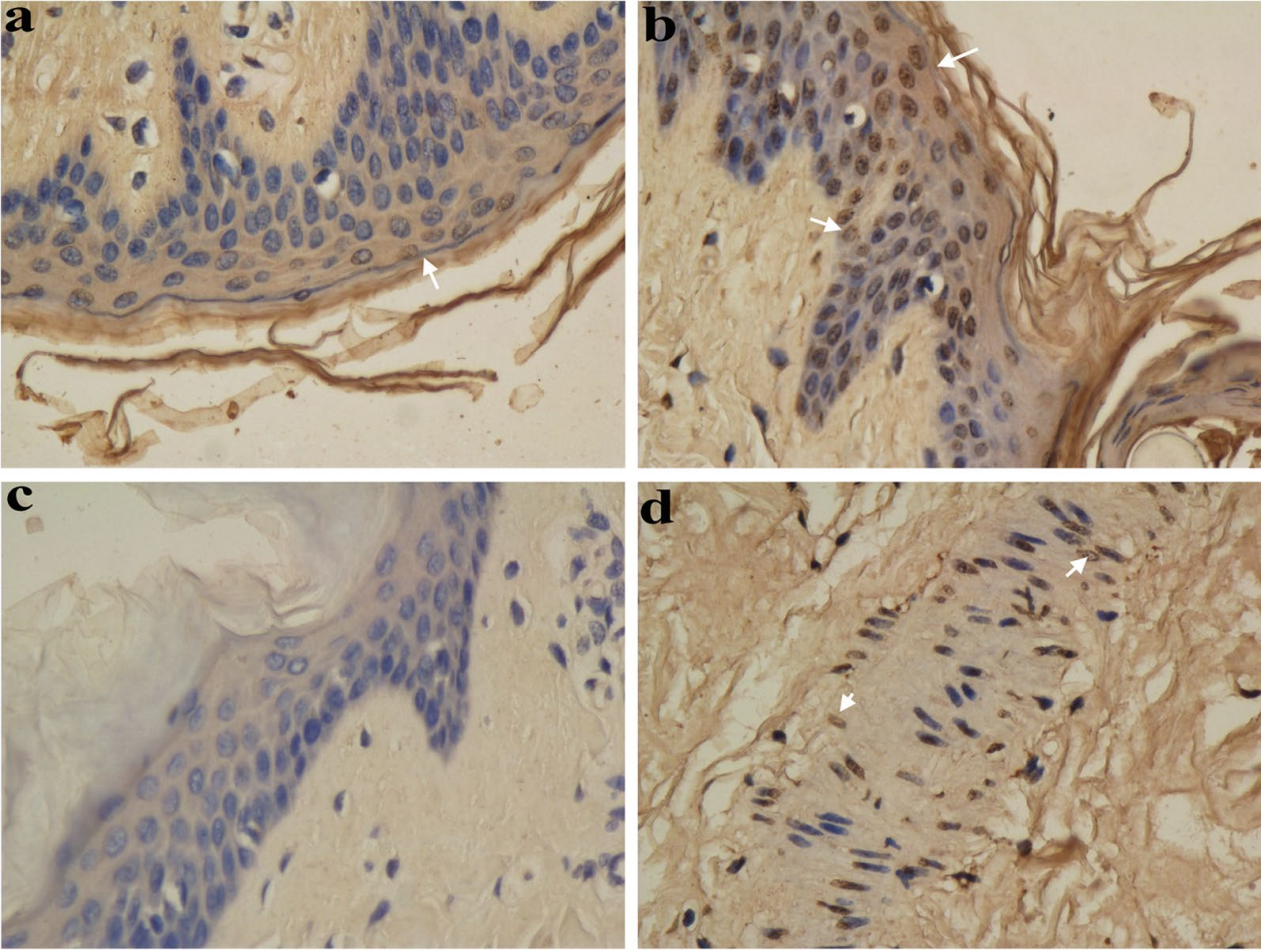
Apoptosis detected with TUNEL immunohistochemistry (*white arrow* brown-yellow nuclear staining). **a** (×400) Immediately after IRE, apoptosis of a few cells in the outmost layer of the breast skin squamous epithelial cells was observed. **b** (×400) Three days after IRE, the apoptosis rate increased. **c** (×400) No apoptosis of the breast skin squamous epithelial cells in the control group was observed. **d** (×400) Apoptosis of vascular smooth muscle cells in the breast

Within three days of IRE, caspase-3-positive staining in the cytoplasm was detected in the ablation areas (Fig. 5a, b). For the pigs in the IRE group, positive TUNEL staining was detected in the nuclei of acinar cells, ductal epithelial cells, adipocytes, stromal cells, vascular endothelial cells and vascular smooth muscle cells in the breast (Fig. 5c, d), and the number of positively stained cells gradually increased in a time-dependent manner, with 100 % of cells undergoing apoptosis by the third day. No cell apoptosis was detected in the sham treatment group or the control group. One week after IRE, there was negative staining in the breast and above the skin (Fig. 5e–h). Apoptosis of vascular endothelial cells and vascular smooth muscle cells in the breast was also detected in the IRE group (Fig. 6). Two hours after IRE ablation, a few apoptotic cells were visible in the outermost layer of epidermal squamous epithelial cells, and the number of apoptotic cells increased on the third day (Fig. 6a–d). Fourteen days after IRE, no apoptotic cells were detected in any of the breasts.

### Imaging

**Fig. 7:**
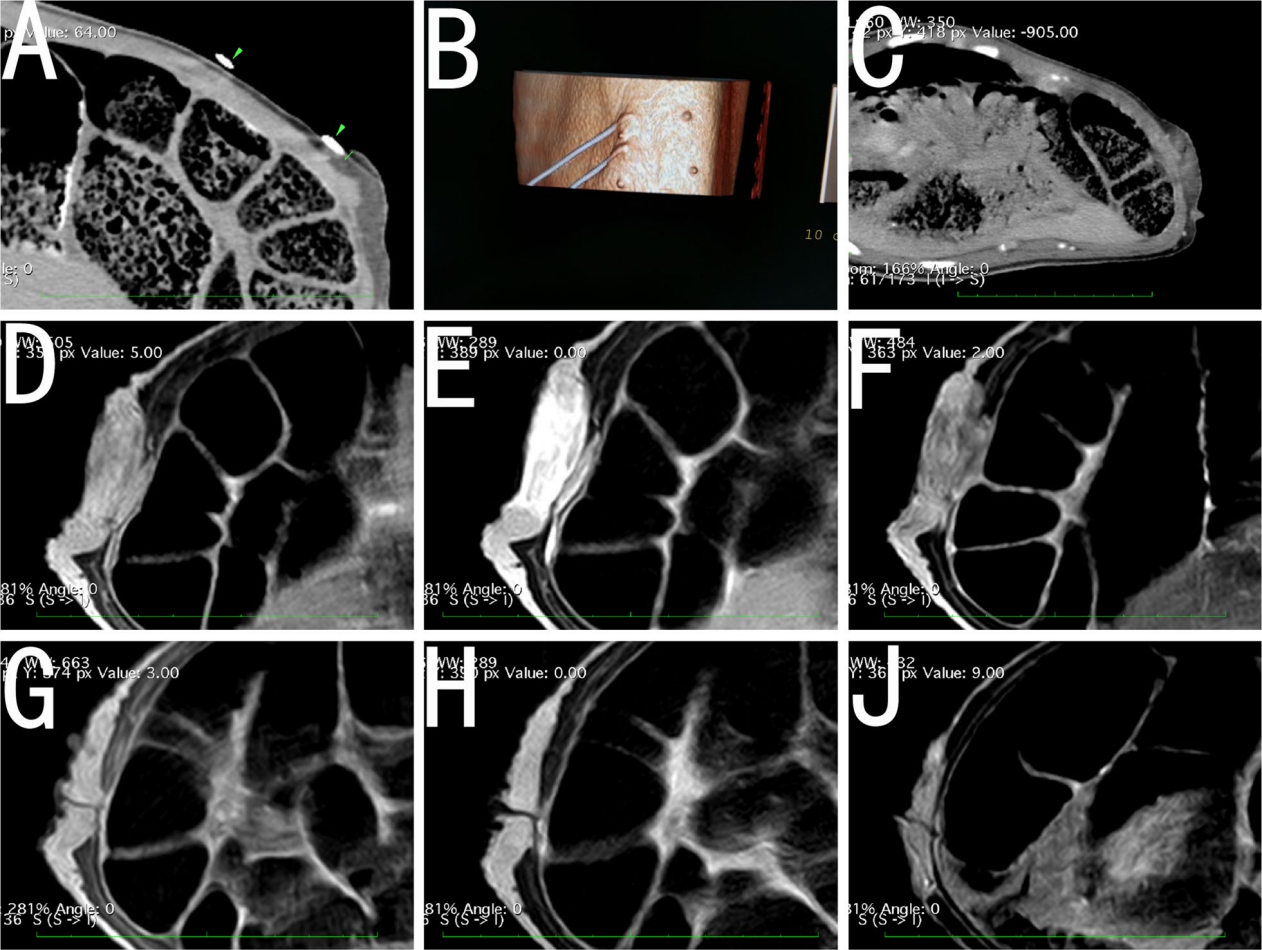
**a** IRE ablation of pig breast (*two arrows*) and measurement of the skin–electrode distance. **b** CT reconstruction of pig breast during IRE with parallel electrodes. **c** Immediately after IRE, there was contrast enhancement and swelling in the breast. **d** Two hours after IRE, T1WI MR imaging showed that the breast was swelling, which was indicated by the low signal intensity. **e** Two hours after IRE, T2WI MR imaging showed that the breast was swollen, with high signal intensity. **f** Two hours after IRE, contrast-enhanced T1WI MR imaging showed the breast was swelling with slight inhomogeneous enhancement. Three months after IRE, T1WI(G), T2WI(H) and contrast-enhanced T1WI(J) MR imaging showed that the breast, skin and nipple of the pig were normal

**Fig. 8:**
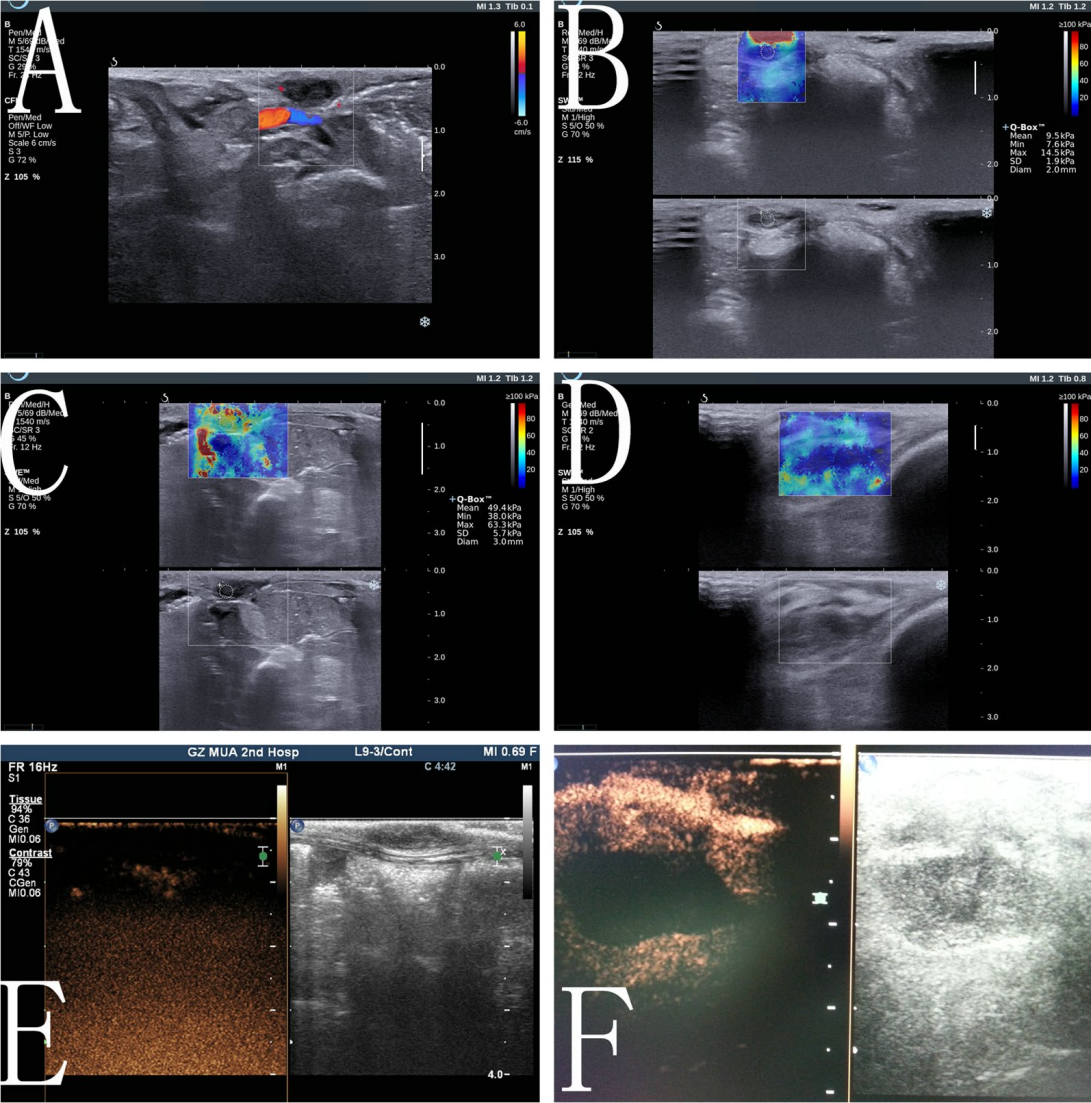
Contrast-enhanced ultrasound and elastosonography before and after IRE ablation of breast cancer in a rabbit model. **a**, **b** Ultrasound and elastosonography before IRE showed the echo and rigidity of the ablation zone. **c**, **d** After IRE, elastosonography led to an increase in the rigidity value (1 day after IRE), followed by a decrease (7 days after IRE). **e** Based on contrast-enhanced ultrasound after IRE, there was no enhancement in the ablation zone (1 day after). **f** Based on contrast-enhanced ultrasound, 7 days after IRE, the area without blood supply had enlarged

Immediately after IRE, the ablated breasts was congested, swollen and enlarged, as confirmed by CT (Fig. 7a–c) and MR imaging (Fig. 7d–j), whereas the ablation zones were hypoechoic according to ultrasound assessment. One week after IRE, CT imaging showed the presence of mild hyperemia and edema in the pig breasts. After 2 months, ultrasounds of the ablated breast areas appeared normal. After IRE, the complete hierarchical architecture of the breast skin and breast tissue was observed using CT (Fig. 8d); and the integrity of the breast architecture appeared normal without scars, cysts or mass formation based on ultrasound. After 3 months, each pig showed a normal breast, skin and nipple appearance (Fig. 7g–j).

Immediately after IRE ablation, ultrasound assessment indicated that the breast tumors in the rabbit model were hypoechoic without contrast enhancement in the ablation zones (Fig. 8a). Elastosonography showed that the tumor elasticity value had increased (Fig. 8b, c). Seven days after IRE, the tumors were still hypoechoic without contrast enhancement, and the elasticity value determined by elastosonography had decreased (Fig. 8d). All of the breast tumors treated with IRE exhibited complete responses, which were confirmed using contrast-enhanced ultrasound (Fig. 8e, f) and histopathology.

After IRE, the targeted breast tissues and tumors were completely inactivated, which was demonstrated using imaging and histopathology, and the only change seen for most breast skin was an alteration in color. Elastosonograhpic measurements showed that the elasticity of the breast tumors significantly increased after IRE, which is consistent with the pathological findings of hyperemia and tumor edema.

## Discussion

Based on gross pathological, histopathological and imaging (ultrasound/CT/MRI) results, we found that most of the experimental animals had well-preserved breast skin, and complete ablation of the targeted zones (including breast tissue and breast cancer) was achieved. HE staining, apoptosis assays, color Doppler ultrasound, contrast-enhanced ultrasound and ultrasound elastography demonstrated the inactivation of breast tumors after IRE. The first day after IRE, the stiffness of the breast neoplasms significantly increased according to elastosonography, which is consistent with the pathological findings of hyperemia, edema and stiffness in the breast tumors. Normal breast tissue regenerated after IRE ablation of breast cancer in the rabbit model, without mass formation or obvious fibrosis. None of the animals died during IRE.

The number of apoptotic cells in each ablation zone increased gradually, reaching a peak on the 3rd day, at which point all cells in each ablation zone were apoptotic. Following IRE ablation of goat liver, Liu et al. [13]. found that the majority of apoptosis occurred 24 h after IRE, but there was no detectable apoptosis around the electrode. Apoptosis duration might vary depending on the equipment and parameters used. These results are consistent with those obtained from another study [14]. Furthermore, it is notable that, in addition to a slight thermal effect in a small region around the electrode, IRE mainly affected the central area between the electrodes. Another study [15] reported that the average ablation-zone cell mortality rate was 77 % at 6 h after IRE and 98 % after 24 h.

After IRE, the apoptotic cells that were observed in the breast tissues were completely absorbed, and normal breast tissue gradually regenerated without fibrosis or mass formation. The ablated breasts subsequently became normal in appearance. This regeneration indicates that structural and functional recovery of the breast had occurred. Because no additional breast masses developed, there was no need to discriminate between treatment-related fibrosis and residual tumor. If these findings are clinically translatable, this would avoid placing an additional psychological burden on the patient. Some researchers have demonstrated the efficacy of IRE ablation against breast cancer cell lines in vitro [11, 16, 17], but skin and duct damage have not been reported. We observed the patency of the mammary duct in this study.

We used different electrodes—monopolar electrode in the study, and we believe it is more suitable for breast cancer ablation in clinical situation. Robert et al. [12] used bipolar electrode for small breast cancer (less than 1 cm in diameter), but the ablation volume was limited and incomplete ablation occurred in two mice. We used monopolar electrodes, which is suitable for breast tumor more than 1 cm in diameter with large ablation volume. Ablation with multiple monopolar electrodes may full cover the tumor, realizing conformal and precise ablation. Also, the State Food and Drug Agency in China usually recommend rabbit and pig for skin irritation study, not mice. By using two or more monopolar electrodes, we input electrodes around the tumor. If we use bipolar electrode and insert it in the tumor, it may bring tumor cells adhered to the electrode and cause tumor implantation. In this study, monopolar electrodes may lessen the chances of cancer cells adhesion to the electrodes and dissemination.

Most of the pig breasts showed no obvious damage to the skin architecture after IRE. This phenomenon might be explained by the non-simultaneous apoptosis of skin squamous epithelial cells and the fast regeneration of these cells; additionally, there was no damage to the collagen architecture of the skin. After performing IRE on pig breast skin at a skin–electrode distance of 1 or 3 mm, the skin color changed; however, this was later completely restored to normal, although an occasional infection was observed. When the skin–electrode distance was 5 mm or more, there was no obvious skin damage and no purulent skin infection in any of the animals. Erythema in breast skin indicates obvious hyperemia in IRE-ablated tissue. The breast skin then changes to a purple color because red blood cells leak out of capillaries and are deposited in and around the ablation zone. Next, hemoglobin gradually transforms into deoxyhemoglobin, and oxygen is released. Thus, we deduced that the observed changes in breast skin color were related to changes in blood supply in the ablation zone. The skin preservation achieved and the lack of mass formation together support the utility of breast cancer ablation.

Recent studies have reported no significant difference in overall survival between breast-conserving surgery and radical mastectomy for specific breast cancers, which provides theoretical support [1] for the use of local ablation to treat breast cancer. After thermal ablation of breast cancer, independent of supplementary postoperative radiotherapy, some breast masses will form and slowly resolve, causing some patients to experience anxiety [2] and potentially leading to infection or fibrotic mass formation in the breast. Skin injury and scar formation are also limited when using radiotherapy and repeated thermal ablation for breast cancer, particularly when the tumor being treated is superficial. Previous reports [2] have shown damage to the skin and pectoralis major after breast ablation, and it can be difficult to differentiate between a scar and a residual tumor or a recurrence [18].

The IRE electrode has a 19G diameter, which helps reduce puncture injury and hemorrhage during breast ablation. However, during IRE, two small electrodes are placed in parallel with a corresponding angle and depth, which might increase the puncture rate and lead to damage. In this study, there were two cases of skin infection in the electrode tract, which might have resulted from mechanical injury and possible thermal damage [19] around the electrode ablation zone. Furthermore, disinfecting the animals was difficult, especially when an ablation zone was located near a fold in the skin. Skin burns are one of the most common complications after thermal ablation [20], although if the IRE parameters are well controlled, thermal damage can be avoided [21, 22].

After the thermal ablation of a breast tumor, the lesion became hyperechoic, which affects efficacy judgment and real-time monitoring. Although cryoablation probes are relatively small and ice ball formation can be monitored using ultrasound, this approach can also activate anti-tumor immunity [23]. High-intensity focused ultrasound also has some disadvantages, including that it is time-consuming, increases the risk of target motion due to pain and discomfort (which could affect treatment accuracy and cause damage to surrounding sensitive structures), and is expensive [24]. However, after IRE, the ablation area becomes hypoechoic, facilitating real-time ultrasound-based monitoring during breast ablation.

In our studies, lesion size and shape were determined by elastography and contrast-enhanced ultrasound, and these parameters were well correlated with the area of cell apoptosis determined by pathology. Contrast-enhanced ultrasound can be used to assess whether the complete ablation of a breast cancer has been achieved by detecting the blood supply in the ablation zone, as reported in many previous studies [25, 26]. Immediately after IRE ablation for breast cancer, increased tissue stiffness, assessed using elastography, was indicative of edema, congestion and cell death. Elastography might complement the use of contrast-enhanced ultrasound to monitor IRE [27]. Timely assessment of IRE ablation might also reduce the possibility of a residual tumor. The presence of mild hyperemia and edema in the pig breast, as well as complete ablation (Additional file 1) of the targeted tissue and breast recovery, were also demonstrated on contrast-enhancement CT and MRI in our study.

## Conclusions

Our study demonstrated that IRE ablation of breast tissue and breast cancer is feasible and safe. After the complete ablation of breast tissue and breast cancer with IRE, the skin architecture was well preserved at a skin–electrode distance of ≥5 mm. In addition, rapid dissipation of apoptotic tissue and breast regeneration occurred in the experimental animals without massive fibrosis or mass formation.

IRE is a potential option for the ablation of breast cancer, particularly for superficial tumors that are in close proximity to susceptible skin.

Due to anatomical differences between humans and animals, clinical trials will be necessary to further assess the skin changes that occur after IRE ablation of breast cancer.

## Additional file

10.1186/s12967-016-0993-7 The setting voltage and current were successfully delivered after IRE, which means irrecoverable pores in the cell membrane were created—a sign of successful ablation of targeted volume.

## Abbreviations

IRE: irreversible electroporation
CT: computed tomography
MRI: magnetic resonance imaging
ECG: electrocardiogram
HE: hematoxylin-eosin staining
TUNEL: terminal deoxynucleotidyl transferase dUTP nick end labeling
