# Supplementary figures and images for: Percutaneous irreversible electroporation for breast tissue and breast cancer: safety, feasibility, skin effects and radiologic–pathologic correlation in an animal study
Source: J Transl Med. 2016 Aug 5;14:238. doi: 10.1186/s12967-016-0993-7 (PMC4975887; doi:10.1186/s12967-016-0993-7)

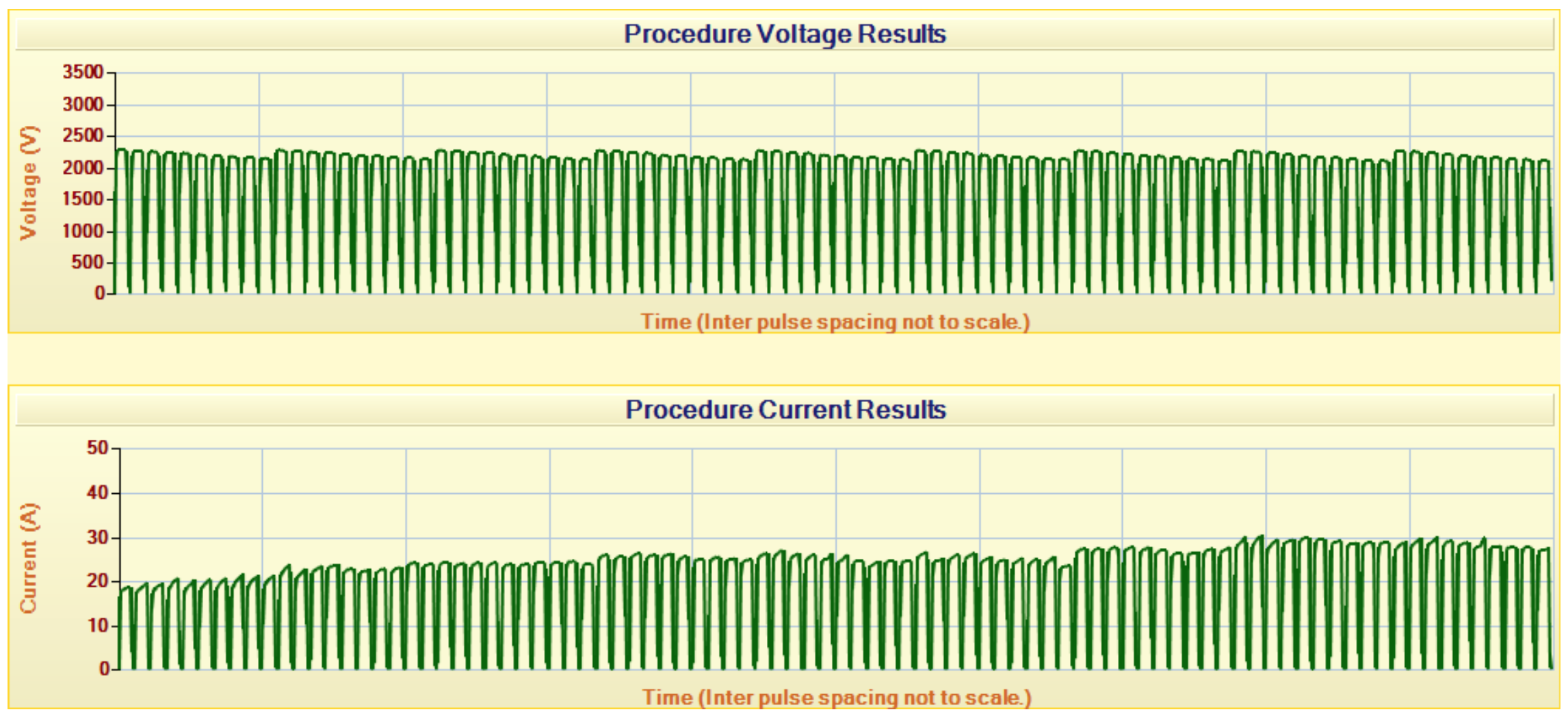

Supplement: Supplementary file 1 — 10.1186/s12967-016-0993-7 The setting voltage and current were successfully delivered after IRE, which means irrecoverable pores in the cell membrane were created—a sign of successful ablation of targeted volume. [file 12967_2016_993_MOESM1_ESM.png]
